# Inhibition of Angiogenic Factor Production from Murine Mast Cells by an Antiallergic Agent (Epinastine Hydrochloride) In Vitro

**DOI:** 10.1155/2008/265095

**Published:** 2008-08-19

**Authors:** K. Asano, A. Furuta, K. Kanai, S. Sakaue, H. Suzaki, T. Hisamitsu

**Affiliations:** ^1^Department of Physiology, School of NRS, Showa University, 1865 Toka-Ichiba, Midori-ku, Yokohama 226-8555, Japan; ^2^Department of Otolaryngology, School of Medicine, Showa University, Shinagawa-ku, Tokyo, Japan

## Abstract

Angiogenesis is an important event both in the development of allergic inflammatory responses and in the pathophysiology of tissue remodeling in allergic diseases. In the present study, therefore, we examined the influence of antihistamines on angiogenesis through the choice of epinastine hydrochloride (EP) and murine mast cells in vitro. Mast cells (5 × 10^5^ cells/mL) presensitized with murine IgE specific for ovalbumin (OVA) were stimulated with 10 ng/mL OVA in the presence of various concentrations of EP for 4 hours. The levels of angiogenesis factors, keratinocyte-derived chemokine (KC), tumor necrosis factor-*α* (TNF), and vascular endothelial growth factor (VEGF) in culture supernatants, were examined by ELISA. We also examined mRNA expression for the angiogenesis factors by RT-PCR. EP significantly inhibited the production of KC, TNF, and VEGF induced by IgE-dependent mechanism at more than 25 ng/mL. Semiquantitative analysis using RT-PCR showed that EP also significantly reduced mRNA expressions for KC, TNF, and VEGF. These results strongly suggest that EP suppresses angiogenesis factor production through the inhibition of mRNA expression in mast cells and results in favorable modification of clinical conditions of allergic diseases.

## 1. INTRODUCTION

Allergic rhinitis (AR) is well accepted to be a symptomatic disease of the nasal mucosa caused by an IgE-mediated allergic inflammation, and characterized by nasal itching, sneezing, water rhinorrhea, and nasal obstruction, which makes breathing through the nose difficult [1]. These clinical symptoms are also well known to be mediated by several factors such as histamine, prostaglandins, and other inflammatory mediators (e.g., inflammatory cytokines) secreted from activated inflammatory cells including eosinophils, mast cells, and T cells in the local of inflammation [1]. In addition to these classical immune responses, structural changes within the nasal walls have also been reported in patients with allergic rhinitis. These structural changes include epithelial disruption, mucus gland hypertrophy, mucosal myofibroblast transformation, and increased matrix protein deposition [2, 3]. These cellular changes are now called tissue remodeling and two groups of proteins matrix metalloproteinases (MMPs) and their counter-regulatory inhibitors, TIMPs, are generally accepted to be important factors for tissue remodeling [4].

Recently, there is increasing evidence that angiogenesis plays an important role in both the development of inflammation and in the pathophysiology of tissue remodeling during allergic responses [2, 5, 6]. Numerous numbers of inducers of angiogenesis have been identified, including vascular endothelial growth factor (VEGF), angiogenin, transforming growth factor (TGF), tumor necrosis factor-*α* (TNF) and interleukin (IL)-8, and others [6, 7]. Some experimental evidence strongly suggests that in inflammation, infiltrating inflammatory cells and some resident cells are the producers of the angiogenic factors. Human neutrophils [8] and T-lymphocytes [9] synthesize and secret the angiogenic factors such as VEGF and IL-8. Peripheral blood eosinophils were found to secrete the factors when stimulated with granulocyte-macrophage colony stimulating factor and IL-5 in vitro [10]. Fibroblasts, as resident cells, are also a demonstrated rich source of the angiogenic factors [11]. Among these cells, mast cells and eosinophils have been highlighted as the effector cells in angiogenesis during allergic inflammation [7, 12]. Although recent researches have focused on the ability of antihistamines, which are the most important agent in the treatment of allergic diseases including AR, to modulate the release of inflammatory cytokines from mast cells and eosinophils, there is little information regarding the effects of antihistamines on angiogenesis. The present study, therefore, was undertaken to examine the influence of epinastine hydrochloride (EP), the most famous antihistamine in Japan, on keratinocyte-derived chemokine (KC), TNF, and VEGF that are known to be major factors affecting angiogenesis [13] in murine mast cells in IgE-dependent manner.

## 2. MATERIALS AND METHODS

### 2.1. Mice

Specific pathogen-free BALB/c male mice were purchased from Charles River Japan Inc. (Atsugi, Japan).

### 2.2. Agents

EP was kindly donated by Nihon Boehringer Ingelheim Co. Ltd. (Tokyo, Japan) as a preservative-free pure powder. This was dissolved in RPMI-1640 medium (SIGMA-ALDRICH Inc., St Louis, MO, USA) supplemented with 10% heat inactivated fetal calf serum from GIBCO BRL (Gaithersburg, Md, USA; RPMI-FCS) at a concentration of 10 mg/mL, sterilized by passing through 0.2 *μ*m filter and then diluted with RPMI-FCS at appropriate concentrations for experiments.

### 2.3. Preparation of mouse IgE

BALB/c mice were injected intraperitoneally twice with a 2-week interval with 5.0 mg of ovalbumin from WAKO Pure Chemicals (OVA; Osaka, Japan) absorbed with 0.5 mg of alum in a volume of 0.5 mL. After one week, blood was obtained by cardiac puncture and OVA specific IgE was purified with KAPTIV-AE from Tecnogen S.C.p.A. (Monte Verna, Italy) according to the manufacturer's instructions. The protein concentration of the extracted solution was measured with protein assay kit from Bio-Rad Laboratories (Hercules, Calif, USA) and adjusted to 1.0 mg/mL with RPMI-FCS.

### 2.4. Preparation of mast cells

Mouse peritoneal mast cells were isolated as previously described [13]. Briefly, mice were anesthetized with diethyl ether and exanguinated by decapitation. The peritoneal cavity was rinsed with 10 mL of heparinized (10 IU/mL) calcium and magnesium-free Hank's solution (HBSS). The fluid was collected, centrifuged at 150 g for 15 minutes at 4°C. The pelleted cells were resuspended in HBSS containing 0.1% bovine serum albumin and submitted to a continuous isotonic Percoll gradient (72%) for mast cell isolation. Purified mast cells were resuspended in RPMI-FCS. The cell purity (>96%) and viability (>98%) were evaluated by alcian blue and trypan blue exclusion staining, respectively.

### 2.5. Sensitization and treatment of mast cells

To sensitize mast cells with IgE, 1 × 10^6^, cells were incubated with 500 ng/mL of OVA-specific IgE for 30 minutes at 37°C, washed, and resuspended in RPMI-FCS. The sensitized mast cells (5 × 10^5^ cells/mL) were stimulated with 10 ng/mL OVA for 4 hours in the presence of various concentrations of EP in a total volume of 2.0 mL. The culture supernatants were collected after centrifugation of cell suspension at 150 g for 15 minutes at 4°C and stored at −40°C prior to assay for factors. To obtain water-soluble intracellular contents, the pelleted cells suspended in 2.0 mL HBSS were then sonically disrupted in an ice cold water bath for 5 minutes and centrifuged at 3000 g for 30 minutes at 4°C. The supernatants were obtained and stored at −40°C until used. In cases of examining mRNA expression, mast cells sensitized IgE were stimulated with OVA in a similar manner for 4 hours and stored at −80°C until used. In all experiments, EP was added to cell cultures one hour before starting antigenic stimulation and cells were cultured in triplicate.

### 2.6. Assay for factors

KC, TNF, and VEGF levels in culture supernatants were assayed using commercially available mouse ELISA test kits (R & D systems, Minneapolis, Minn, USA) according to manufacturer's recommendation.

### 2.7. Real-time polymerase chain reaction

mRNA was extracted from mast cells using *μ*MACS mRNA isolation kits from Miltenyi Biotec GmbH (Bergisch Gladbach, Germany) according to the manufacturer's instructions. The first-strand complementary DNA (cDNA) synthesis from 1.0 *μ*g mRNA was performed using the SuperScript Preamplification System for cDNA synthesis from GIBCO BRL (Gaithersburg, Md, USA). PCR was then carried out using a GeneAmp 5700 Sequence Detection System from Applied Biosystems (Foster City, Calif, USA). PCR mixture consisted of 2.0 *μ*l of sample cDNA solution (10.0 ng/mL), 25.0 *μ*l of SYBR-Green Mastermix (Applied Biosystems), 0.3 *μ*l of both sense and antisense primers, and distilled water to give a final volume of 50.0 *μ*l. The reaction was conducted as follows: 4 minutes at 95°C, followed by 40 cycles of 15 seconds at 95°C, and 60 seconds at 60°C. *β*-actin was amplified as an internal control. mRNA levels were calculated by using the comparative parameter threshold cycle (Ct) and normalized to *β*-actin. Oligonucleotide sequences of the primers used are as follows: for KC [14], 5′-GCGCCTATCGCCAATGAG-3′ (sense) and 5′-AGGGCAACACCTTCAAGCTCT-3′ (antisense); for VEGF [15], 5′-CAGCTATTGCCGTCCGATTGAGA-3′ (sense) and 5′-TGCTGGCTTTGGGAGGTTTGAT-** ** **3′ (antisense); for TNF [16], 5′-CCTGTAGCCCACGTCGTAGC-3′ and 5′-TTGACCTCAGCGCTGAGTTG-3′; for *β*-actin [16], 5′-ACCCACACTTGTGCCCATCTA-3′ (sense) and 5′-CGGAACCGCTCATTGCC-3′ (antisense).

### 2.8. Statistical analysis

The statistical significance of the data between the control and experimental groups was analyzed by ANOVA followed by Fisher's PLSD test. *P* < .05 was considered statistically.

## 3. RESULTS

### 3.1. Suppressive activity of EP on KC, TNF, and VEGF production from mast cells

**Figure 1:**
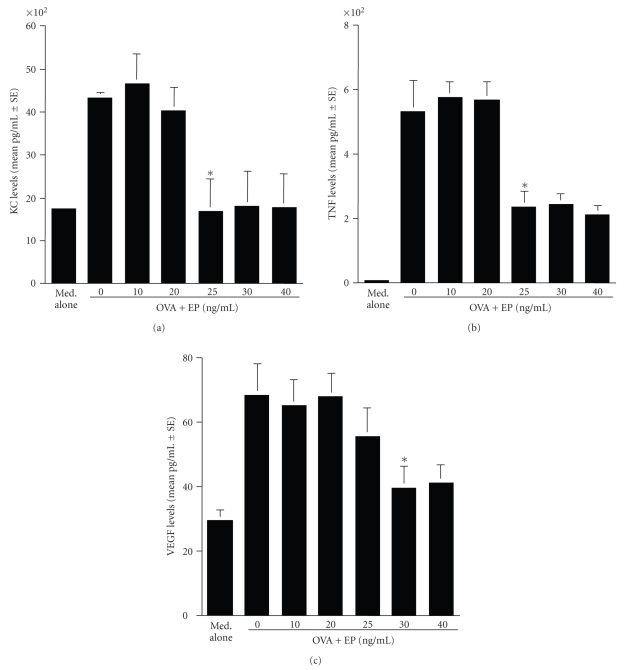
Suppressive activity of epinastine hydrochloride (EP) on angiogenic factor production from mast cells in IgE-dependent mechanism. Murine peritoneal mast cells (5 × 10^5^ cells/mL) sensitized with IgE specific for ovalbumin (OVA) were stimulated with 10 ng/mL OVA in the presence of various concentrations of EP for 4 hours. The levels of angiogenic factors in culture supernatants were analyzed by ELISA. The data were expressed as the mean pg/mL ± SE of triplicate cultures. KC: keratinocyte-derived chemokine (a); TNF: TNF-*α* (b); VEGF: vascular endothelial growth factor (c);*P < .05 versus control (EP at 0 ng/mL).

**Figure 2:**
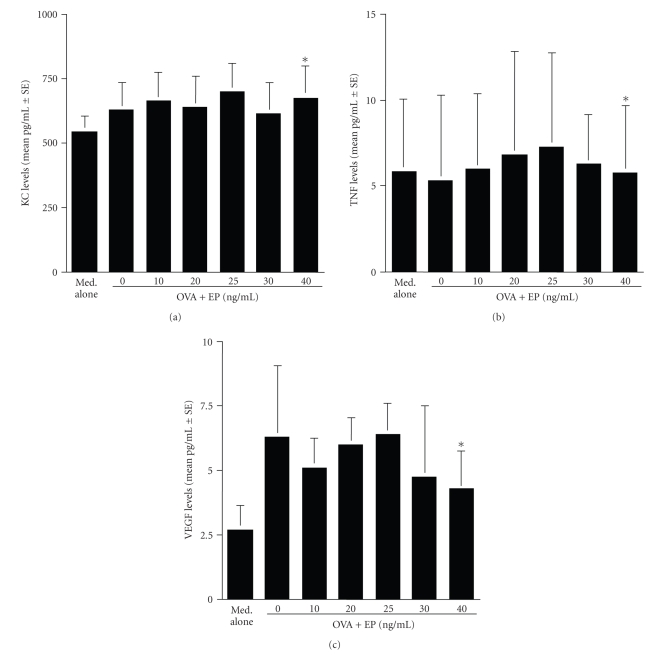
Influence of epinastine hydrochloride (EP) on angiogenic factor release from mast cells in IgE-dependent mechanism. Murine peritoneal mast cells (5 × 10^5^ cells/mL) sensitized with IgE specific for ovalbumin (OVA) were stimulated with 10 ng/mL OVA. After 4 hours, cells were collected, sonically disrupted, and the levels of angiogenic factors in cytosole were examined by ELISA. The data are expressed as the mean pg/mL ± SE of triplicate cultures. KC: keratinocyte-derived chemokine (a); TNF: TNF-*α* (b); VEGF: vascular endothelial growth factor (c);*P < .05 versus control (EP at 0 ng/mL).

The first set of experiments was undertaken to examine the influence of EP on KC, TNF and VEGF production from mast cells induced by antigenic stimulation in vitro. Mast cells (5 × 10^5^ cells/mL) sensitized with OVA specific IgE were stimulated with OVA in the presence of 0, 10, 20, 25, 30, and 40 ng/mL EP for 4 hours. Factor levels in culture supernatants were assayed by ELISA. As shown in Figures 1(a) and 1(b), treatment of cells with EP at quantities lower than 20 ng/mL did not cause the suppression of the release of both KC and TNF, which was increased by antigenic stimulation. However, EP significantly suppressed the ability of cells to release both KC and TNF after antigenic stimulation, when the agent was added to cell cultures at 25 ng/mL and higher (Figures 1(a) and 1(b)). The data in Figure 1(c) also showed the suppressive effect of EP on VEGF release from mast cells induced by antigenic stimulation; treatment of mast cells with EP at more than 30 ng/mL significantly suppressed increase in VEGF levels induced by antigenic stimulation. We then examined factor levels in water-soluble intracellular extracts by ELISA. As shown in Figures 2(a), 2(b), and 2(c), treatment of mast cells with EP did not cause the prevention of factor release from cells; the levels of factors (KC, TNF, and VEGF) in extracts from cells treated with EP at 40 ng/mL are nearly identical (not significant) to that in stimulated but nontreated control (OVA + IgE).

### 3.2. Inhibitory effects of EP on the mRNA expression for KC, TNF, and VEGF

**Figure 3:**
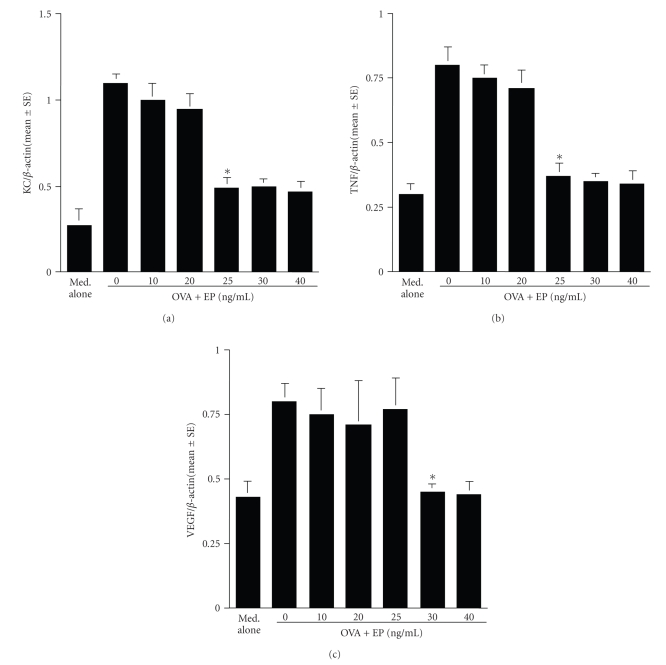
Suppressive activity of epinastine hydrochrolide (EP) on mRNA expression for angiogenic factors in mast cells in IgE-mediated mechanism. Murine peritoneal mast cells (5 × 10^5^ cells/mL) sensitized with IgE specific for ovalbumin (OVA) were stimulated with 10 ng/mL OVA for 4 hours. mRNA expression for angiogenic factors was examined by real-time RT-PCR. The data are expressed as the mean ± SE of triplicate cultures. KC: keratinocyte-derived chemokine (a); TNF: TNF-*α* (b); VEGF: vascular endothelial growth factor (c);*P < .05 versus control (EP at 0 ng/mL).

The second group of experiments were undertaken to examine the influence of EP on mRNA expression for KC, TNF, and VEGF after OVA stimulation. Mast cells (5 × 10^5^ cells/mL) sensitized with OVA specific IgE were stimulated with OVA in the presence of 0, 10, 20, 25, 30, and 40 ng/mL EP for 4 hours. mRNA expression was examined by semiquantitative RT-PCR. As shown in Figures 3(a), 3(b), and 3(c), treatment of mast cells with EP suppressed mRNA expression for factors examined, which was increased by OVA stimulation. The minimum concentration of the agent, which caused significant suppression, was 25 to 30 ng/mL.

## 4. DISCUSSION

EP is a selective and potent H_1_-receptor antagonist with no anticholinergic and sedative effect [17, 18]. Our previous work clearly showed that EP could suppress thymus and activation-regulated chemokine (TARC) production from human peripheral blood T cells induced by IL-4 and costimulatory molecule stimulation [19]. It is also observed that EP could antagonize against IL-4-mediated T cell cytokine imbalance in vitro [20]. These reports strongly suggest that the modulation of EP on both cytokine and chemokine production, which are responsible for the development of allergic immune responses, consists, in part, of a therapeutic mode of action of the agent on allergic diseases.

The structural changes in the airway walls including epithelium, submucosa, smooth muscle, and vasculature are referred to as airway remodeling, which is mainly induced by several types of MMPs, in allergic diseases such as asthma and allergic rhinitis [4, 21, 22]. Furthermore, there is accumulated evidence that allergic inflammation causes structural changes in the nasal wall, which is characterized by angiogenesis and subepithelial basement membrane hypertrophy [2, 3, 23, 24]. Angiogenesis involves destruction of the basement membrane by MMPs, migration and proliferation of endothelial cells, and transformation of endothelial cells to form tubes [25]. This process is mediated by numerous inducers, including members of fibroblast growth factor family, VEGF, angiogenin, transforming growth factor, TNF, interleukins and chemokines [7, 26]. Among them, VEGF is the most potent regulator of angiogenesis and induces the enhancement of proliferation, migration, and tube formation of endothelial cells [7, 26]. VEGF promotes secretion of MMP-1 and the expression of chemokines, as well as intracellular adhesion molecule and E-selectin [7]. VEGF also causes vasodilation, which is responsible for edema, inflammatory cell infiltration and increase in nasal secretions, through the induction of the endothelial nitric oxide synthase and the subsequent increase in nitric oxide production [7, 26]. Furthermore, VEGF stimulates monocyte chemotaxis and contributes to the recruitment of bone-marrow-derived endothelial cells in angiogenesis [7]. Together with the present results, showing the suppressive activity of EP on VEGF production may be interpreted as meaning that some of therapeutic effects of EP in AR depend on its suppression of VEGF production from mast cells. In addition to VEGF, TNF is considered to be a multifunctional proinflammatory cytokine that plays a central role in the initiation and maintenance of many inflammatory diseases, including asthma and allergic rhinitis. Although TNF is associated predominantly with Th1-dependent inflammation, recent reports indicate that TNF is essential for the production of the Th2 type cytokines and for infiltration of Th2 T cells into the site of allergic inflammation [27]. TNF is produced by a variety of inflammatory cells, especially mast cells and macrophages through IgE-dependent mechanisms and also enhanced the effect of both IL-4 and IL-10 on antigen-specific IgE production [27, 28]. Furthermore, TNF increased the expression of mRNA for endothelial-leukocyte adhesion molecule-1 (ELAM-1) and vascular cell adhesion molecule-1 (VCAM-1), which are essential for eosinophil migration into allergic inflammatory site, in nasal mucosa after ovalbumin sensitization in mice [27], suggesting that inhibition of TNF production from mast cells through IgE-dependent mechanisms may also partially account for the therapeutic mechanisms of the agent on allergic diseases.

KC (murine homolog of IL-8), a member of CXC subfamily, is produced by several types of immune cells, including epithelial cells, macrophages, mast cells, and lymphocytes after immunological and nonimmunological stimulation [29]. Although several reports clearly showed that the presence of large amount of IL-8, chemoattractants for neutrophils and eosinophils, in nasal lavage fluid obtained from allergic rhinitis [30–32], the role of this chemokine is limited in granulocyte infiltration into inflammatory sites of allergic rhinitis [29]. On the other hand, addition of IL-8 into human microvascular endothelial cells cultured on the surface of a three-dimensional collagen gel-caused-tube-like structure formation [32]. It is also reported that administration of anti-IL-8 into rabbit corneas significantly inhibits newly vessel formation (angiogenesis) induced by implantation with ethylene vinyl acetate pellets containing TNF [33], indicating that IL-8 plays an essential role in TNF-mediated angiogenesis. From these reports, the inhibitory action of EP on KC production from mast cells by IgE-dependent mechanisms may also have important therapeutic implications for allergic diseases.

Although the present results clearly show the inhibitory action of EP on the production of proangiogenic factors from mast cells by antigenic stimulation through the suppression of mRNA expression, the precise mechanisms by which EP could inhibit the production of proangiogenic factors from mast cells are not clear at present. The major stimulus for mast cell activation is the aggregation of the high affinity receptor for IgE so-called Fc*ε*RI [34]. Crosslinking of Fc*ε*RI-bound IgE-molecules with specific allergen activates the receptor-associated tyrosine kinases and the initiation of further phosphorylation of downstream molecules such as Syk, phosphatidyl-inositol-3-OH-kinase, and phospholipase C*γ* [34]. These proximal signaling events activate protein kinase C that ultimately initiates mast cell effector functions such as degranulation and cytokine production [34]. The activation of these kinases is also reported to require Ca^2+^, which is increased by crosslinking of IgE with allergen on mast cell surface [35]. It has been reported that EP at more than 10^−5^ M could inhibit Ca^2+^ influx and Ca^2+^ release from the intracellular calcium store of mast cells exposed to compound 48/80 and substance P [36, 37]. Judging from these reports, it is strongly suggested that EP inhibits the changes in Ca^2+^ concentration in cytosole induced by antigenic stimulation and results in suppression of angiogenic factor production from mast cells. In addition to these signaling pathways, transcription factor NF-*κ*B regulates the expression of inflammatory gene products in many different cell lineages. In mast cells, the expression of TNF, IL-6, and IL-8 gene after Fc*ε*RI ligation strictly depends on the activation of NF-*κ*B [34, 35]. NF-*κ*B is a ubiquitous protein transcription factor and normally resides in an inactive state in cytoplasm. However, when activated it translocates to the nucleus, binds the DNA, and activates genes. Activation of NF-*κ*B is induced by degradation and dissociation of I*κ*B, an endogenous inhibitor of NF-*κ*B, through Ca^2+^-dependent and -independent mechanisms [20]. Together with the present results, there is also possibility that EP inhibits NF-*κ*B activation through Ca-independent mechanisms and results in suppression of angiogenic factor production from mast cells.

Pharmacological studies have revealed that, after oral administration of EP into patients with allergic diseases at a single dose of 20 mg, plasma concentration of this agent was gradually increased and attains a plateau at 26.9 ± 9.1 ng/mL [38], suggesting that the findings of the present in vitro study may reflect the biological function of EP in vivo.

In conclusion, the present results demonstrate that the inhibitory action of EP on angiogenic factor production consists, in part, of a therapeutic mode of action of the agent on allergic diseases, including atopic allergy and pollinosis.
